# Role of viral and host factors in interferon based therapy of hepatitis C virus infection

**DOI:** 10.1186/1743-422X-10-299

**Published:** 2013-10-01

**Authors:** Muhammad Imran, Sobia Manzoor, Javed Ashraf, Madiha Khalid, Muqddas Tariq, Hafiza Madeha Khaliq, Sikandar Azam

**Affiliations:** 1 Atta-ur-Rahman School of Applied Biosciences, National University of Sciences and Technology (NUST), 44000 Islamabad, Pakistan; 2 Islam Dental College Sialkot, Sialkot, Pakistan

**Keywords:** Hepatitis C, Antiviral, Interferon, Host factors, Single nucleotide polymorphism, Responders

## Abstract

The current standard of care (SOC) for hepatitis C virus (HCV) infection is the combination of pegylated interferon (PEG-IFN), Ribavirin and protease inhibitor for HCV genotype 1. Nevertheless, this treatment is successful only in 70-80% of the patients. In addition, the treatment is not economical and is of immense physical burden for the subject. It has been established now, that virus-host interactions play a significant role in determining treatment outcomes. Therefore identifying biological markers that may predict the treatment response and hence treatment outcome would be useful. Both IFN and Ribavirin mainly act by modulating the immune system of the patient. Therefore, the treatment response is influenced by genetic variations of the human as well as the HCV genome. The goal of this review article is to summarize the impact of recent scientific advances in this area regarding the understanding of human and HCV genetic variations and their effect on treatment outcomes. Google scholar and PubMed have been used for literature research. Among the host factors, the most prominent associations are polymorphisms within the region of the interleukin 28B (IL28B) gene, but variations in other cytokine genes have also been linked with the treatment outcome. Among the viral factors, HCV genotypes are noteworthy. Moreover, for sustained virological responses (SVR), variations in core, p7, non-structural 2 (NS2), NS3 and NS5A genes are also important. However, all considered single nucleotide polymorphisms (SNPs) of IL28B and viral genotypes are the most important predictors for interferon based therapy of HCV infection.

## Introduction

HCV infects about 200 million people worldwide [1]. Approximately 20–30% of patients naturally clear the virus. About 70–80% acute HCV infections become chronic which leads to the development of cirrhosis in 20% of cases while the same percentage of those patients becomes a victim of hepatocellular carcinoma. Acute hepatitis C occurs during the first six months of HCV infection [2]. Approximately 70-80% of acute hepatitis C cases are without symptoms and hence difficult to diagnose. The remaining 20%-30% of cases are associated with symptoms such as pain in joints and muscles, pain in the right upper quadrant, poor appetite, nausea, vomiting, and fever. The case of acute hepatitis C infection is converted into a chronic disease, if the individual is not capable of clearing the virus within few months of infection [3]. Being the member of the *Flaviviridae* family of viruses, HCV is a single stranded RNA virus. Its size is 55 – 65 nm [2]. In 1989 it was realized that HCV is the cause of most transfusion-associated non-A and non-B hepatitis infections. There are about eleven different genotypes of HCV with various subtypes and strains [3]. The virus encodes a poly-protein of 3010 amino acids which is processed to generate four structural (Core, E1, E2 and P7) and six non-structural (NS2, NS3, NS4A, NS4B, NS5A, NS5B) proteins [4]. Recently, great efforts have been made to develop interferon free therapy against HCV infection but interferon is still accepted as a part of standard therapy [5]. Therefore, it would be very helpful for clinical practitioners and researchers to get information about viral and host factors that influence interferon treatment of HCV infection.

### Interferon and its signaling pathway

**Figure 1:**
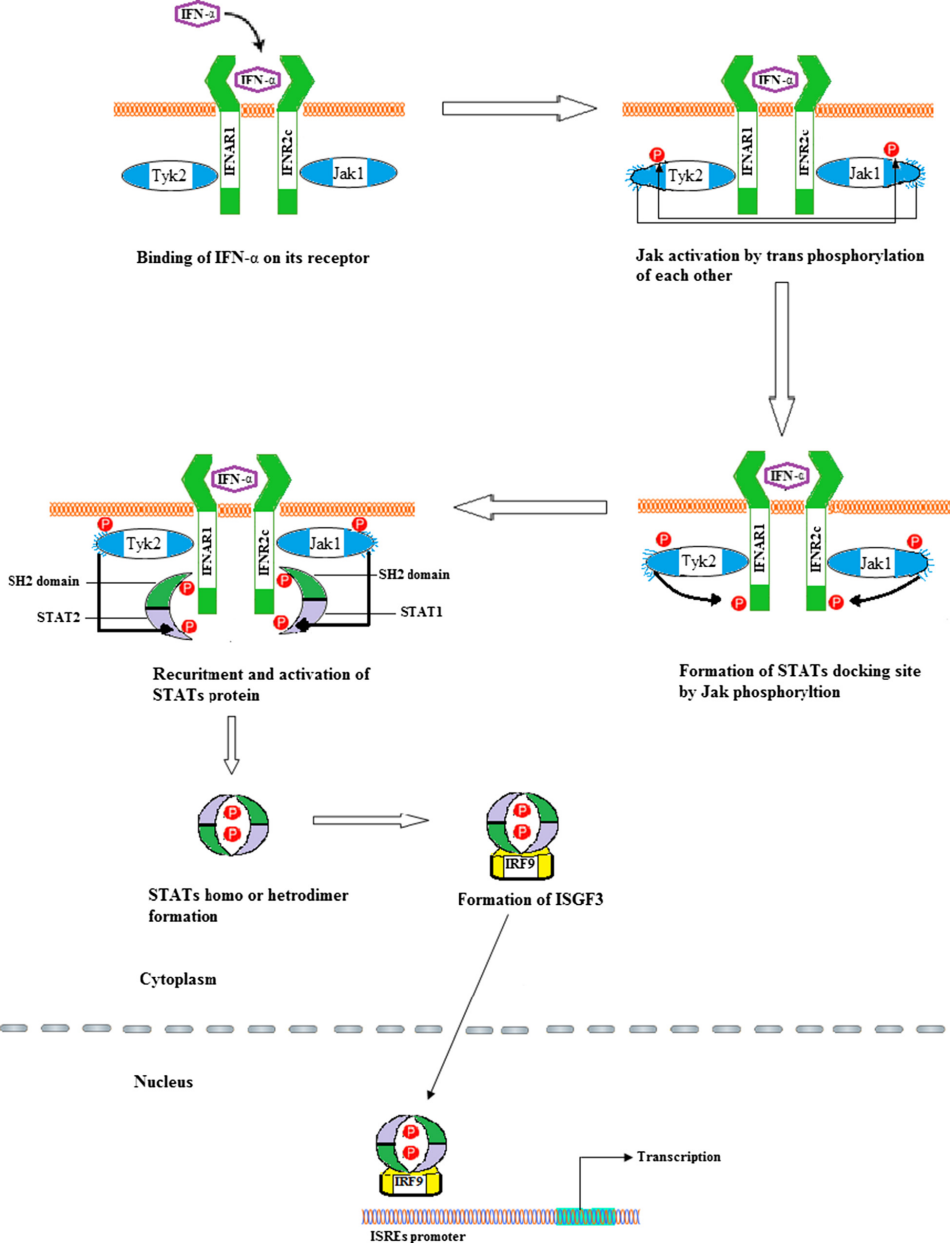
Interferon signaling pathway.

About 50 years ago, IFN was discovered by Isaacs and Lindenmann [6]. Currently, there are about 10 mammalian IFN species with many subspecies. IFN possesses antiviral activity and is categorized into three groups [7]. Type I IFNs include IFN-α, IFN-β, IFN-ϵ, IFN-κ, IFN-ω and IFN-ν. All these interferons interact with the interferon alpha/beta receptor (IFNAR) [8,9].Type II IFN involves only IFN-γ which interacts with a discrete receptor, the interferon gamma receptor (IFNGR) [10]. IFN-λ1, IFN-λ2 and IFN-λ3 are grouped in type III IFNs. These interferons are also known as IL29, IL28A and IL28B respectively. Type III IFNs, signal through IFN-λ receptor which possesses an IL-10R2 chain shared with the IL-10 receptor, and an exceptional IFN-λ chain [11]. IFNAR is the receptor for type I IFNs. It possesses two major subunits: IFNAR1, which bind styrosine kinase 2 (TYK2) [12], and IFNAR2c which binds Janus kinase 1 (JAK1) [9]. Both TYK2 and JAK1 are members of the Jak family. After binding the receptor chains, TYK2 and JAK1 are stimulated and transactivated leading to the initiation of phosphorylation cascades involving all the members of the signaling pathway and also the activators of transcription such as signal transducer and activator of transcription STAT1, STAT2 and STAT3. STAT1, STAT2 and STAT3 are stimulated by type I IFNs in most of cells. STAT1 and STAT2 in combination with another transcription factor, interferon regulatory factor 9 (IRF9), form interferon stimulated gene factor 3 (ISGF3) which binds to the promoter region of interferon stimulated genes (ISGs) as shown in Figure 1. The ISGs are a set of genes used for antiviral protection. Microarray analysis of human and murine cells treated with interferons revealed that there are more than 300 ISGs [13]. Most important of these proteins are the double-stranded RNA-dependent kinase "protein kinase RNA-regulated" (PRKR), the 2′–5′ oligoadenylate synthetases and the Mx proteins. These proteins are known to impede the growth of certain viruses. However, if these genes are knocked out from cells, they still retain their antiviral activities as there are many of other genes stimulated by interferon possessing antiviral activities [14]. Alternatively, STAT1 and STAT3 form homodimers or heterodimers which bind gamma activated sequence (GAS) elements. After binding, STAT proteins activate these genes to generate an antiviral state [15]. Receptors and pathways involved in type III IFNs signaling diverge from those mediating type I IFNs signals. IFN-λ1-3 signal through the JAK/STAT signaling pathway using the IL28-R/IL-10R receptor complex that is mainly expressed on hepatocytes and epithelial cells [16] as opposed to IFNAR that are broadly expressed.

### Ribavirin

Ribavirin (1-b-D-ribofuranosyl-1, 2, 4-triazole-3-carboxamide) inhibits HCV replication. It is a synthetic guanosine nucleoside analogue that inhibits inosine monophosphate dehydrogenase leading to the depletion of the guanosine pool [17]. It is also incorporated into the viral genome and induces error catastrophe by the HCV non-structural-5B (NS5B) polymerase [18]. In addition to these functions, it is thought to have immune-modulatory functions. It regulates macrophages, T helper cells and Th1/Th2 produced cytokines, enhances the expression of interferon stimulated genes (ISG) and also IFN-α and MxA expression.

### Protease inhibitors

Two important NS3 protease inhibitors are Boceprevir and Telaprevir. Boceprevir inhibits NS3 by acting as a non-covalent inhibitor. It inhibits CYP3A4 and acts also as a mild inhibitor of P-glycoprotein. Therefore, the plasma levels of the drugs that are metabolized by CYP3A4 and P-glycoprotein are also increased when given along Boceprevir [19].

## Role of viral factors in determining the response to interferon therapy

### Genotype

Soon after the discovery and sequencing of HCV [20], HCV isolates from different parts of the world with varying sequences were obtained [21,22]. HCV sequences which varied up to 33-36% were classified as genotypes; sequences which varied up to 20 – 27% were classified as subtypes while genetic variants up to 12% within individual isolates were classified as quasi species. The HCV genotype is acknowledged as the most significant independent response marker of interferon therapy [23]. End-of-treatment response (ETR) and SVR are the two important scales for measurement of treatment response. An ETR is referred as undetectable hepatitis C RNA at the end-of-treatment while SVR is referred as undetectable hepatitis C RNA after 24 weeks of treatment [24]. HCV genotypes 2 and 3 are more responsive to interferon therapy than any other HCV genotype [25]. Meta-analyses of IFN-α monotherapy have shown that SVR was obtained in 55% of patients with HCV genotypes 2 and 3 and in 18% of patients with HCV genotype 1 [26]. It is suggested that HCV genotype 6 gives a treatment response similar to genotypes 2 and 3 [24,25]. Although HCV genotyping is an important predictor for the treatment response, no patient should be left without therapy on the basis of the genotype alone [27] because the predictive value of HCV genotyping for interferon based therapy is only 55%.

### 5′UTR

The important role of 5′UTR (Untranslated Region) in translation of HCV polyprotein demands the conservation of this region. There are four highly conserved structural domains in 5′UTR which are numbered I to IV; all of these domains interact with the host factors and are crucial for HCV polyprotein synthesis [28]. Domain III of 5′UTR binds eukaryotic initiation factor 3 (eIF3) by means of stem-loop 3b. It has been suggested that insertions in domain 3 of 5′UTR causes altered Watson-Crick base pairing leading to decreased RNA stability and binding affinity to ribosomal proteins. These mutations were more common in SVR than in breakthrough (BT) patients. Thus, mutations within the domain III of 5′UTR are important for the treatment response in HCV infection [29].

### Variations of core, p7, NS2, NS3 and NS5A

Inter-patient genetic variations within the genotype 1 HCV also exert an important influence on the treatment response. Although sequencing of the HCV genome has not shown any specific amino acid sequences that affect the treatment outcome, the high levels of genetic variations, mostly in the core, p7, NS2, NS3 and NS5A genes, are linked with SVR [30]. Recent data from the Chinese population have also shown that increased genetic variations in the p7, NS2 and NS3 genes of HCV genotype 1b were linked with SVR to the standard treatment [31]. Thus, there is more genetic diversity in HCV SVR patients than in NR patients receiving the standard treatment. Analyses of different HCV sequences identified many specific regions of core, p7, NS2, NS3 and NS5A, which were significantly associated with the ultimate treatment outcome. Among these regions, the most important region was the interferon sensitivity determining region (ISDR) that is located in HCV NS5A [32,33]. The sequence of HCV core region from 50 Swedish patients infected with HCV genotype 1 revealed that substitutions of core residue 70 were associated with poor response to the standard treatment [34]. Moreover, it has been shown that amino-acid (aa) substitution at core residue 70 was the predictor of SVR to a triple therapy of Telaprevir, pegylated interferon and Ribavirin in the Japanese population [35]. How these variations of HCV genome effect response to interferon therapy is still a challenge.

### Role of host factors in determining response to interferon therapy

With the beginning of the human genetic era, it is anticipated that the human genome may significantly influence the clinical management of infectious diseases. But there are small numbers of studies showing the importance of such knowledge in regular clinical practices. HCV infection is one of the most hopeful examples where genetic information was used for therapy. Thus, the treatment response to HCV infection not only depends on the viral factors but also on the host factors. Male sex, older age, insulin resistance, diabetes, African or American ethnicity, cirrhosis, steatosis, and weight (in terms of BMI) are all factors linked to poor response to PEG-IFN plus RBV treatment [36]. Other infections such as HIV, HBV and practices such as alcohol intake and drug use are also responsible for low SVR rates [37]. Currently, candidate gene approaches had been implemented to discover the host factors associated with the HCV treatment response [38]. Following are some of the most important SNPs linked with the treatment response to HCV infection, also shown in Table 1.

**Table 1:** Association of host SNPs with treatment response to HCV infection

**Gene symbol**	**Function**	**SNP**	**Effect on interferon therapy**
**IFN –λ**			
**IL-29 (IFN-λ1)**		rs8099917	TT genotype is favorable
**IL-28A (IFN-λ2)**	Inhibit viral replication	rs12980275	AA genotype is favorable
**IL-28B (IFN-λ3)**		rs12979860	CC genotype is favorable
**IFN - γ**	Inhibit viral replication	-768G	Enhances promoter activity 2-3 folds
**MBL**	Pathogen recognition receptor	O/A at exon 1	X or O mutations linked with non-responsiveness
		At promoter region: MBL2*H,Land X,Y.	
**CTLA4**	Down regulates T cell functions	-318 C/T	-318C, 49G are favorably linked with therapy response.
**IL-10**	Anti-inflammatory, Down regulates MHC1and MHC II molecules	-318 C/T	-819T and -592A are positively associated
		**-**819,	
		**-**592	
**IL-18**	Pro-inflammatory cytokine	607 C/A,	-607A and -137C are positively associated
	Induces IFN-γ	-137 G/C	
**TRAIL**	Induces apoptosis in virally infected cells	rs 4242392	Poorly associated
**TGFb1**	Multifunctional cytokine	codon 10T/C, codon 25G/C	Positively associated
**Mx1**	Antiviral activities	G/T at nt -88	Positively associated
**Osteopontin**	Induces Th1 response	nt 443, nt 1748	T/T at nt -443
			G/G or G/A at 1748
			Positively associated with SVR
**LMP7**	HLA-1 antigen presentation	LMP7-K	Positively associated with SVR
**OAS1**	converts ATP into 2'-5' linked oligomers of adenosine	at exon 7 SAS	AA genotype is poorly associated

## SNPs of host genes

### Interferon-λ

The IFN-λ family was discovered in 2003. The three members of this family, IFN-λ 1, 2, 3, (equivalent to IL-29, 28A, 28B) show a high degree of homology to each other i.e. more than 80%, but very low sequence homology to both IFN-α (15-19% identity, 31-33% similarity) and IL-10 (11-13% identity, 22-23% similarity). Despite this minimal homology, IFN-λ like IL-10 geneare composed of five to six exons [39]–[41]. IFN-λ is produced by many immune cells, neuronal cells, alveolar epithelial cells, hepatocytes, and a variety of cell lines [42]–[44]. However, the primary sources of IFN-λ are dendritic cells (DCs) [42]–[46]. Similar to IFN-α, these cytokines are mainly produced in response to viral infection or by activation of Toll-like receptors (TLRs) [16,47]. IFN-λ showed antiviral activity against many viruses such as Encephalomyocarditis virus (EMCV), vesicular stomatitis virus, cytomegalovirus, herpes simplex virus 1, influenza A virus, HIV, HBV, and HCV [39,40,47]–[49]. These studies revealed that IFN-λ mainly inhibits viral replication but also has immune-modulatory functions. It modulates both the maturation and differentiation of immune cells [50]–[52]. In short, these cytokines have an important role in regulation and development of the adaptive immune response against viruses.

SNPs within chromosome 19, in the vicinity of the IL29, IL28A, and IL28B genes, are importantly associated with the treatment response of HCV infection. Three SNPs related to these genes, rs8099917, rs12980275, and rs12979860, are very important. Previous studies have shown that these variants are significant predictors of the treatment response [53]. The favorable genotypes significantly predicting higher SVR rates are CCrs12979860 irrespective of the race [54], AArs12980275, and TTrs8099917. Nevertheless, the association between IFN-λ production and SNPs in close proximity of IL28 remains indistinct [16,55]. IFN-λ-based drugs are possible candidates for treating HCV infection, and are presently being evaluated in clinical trials. The adverse effects of IFN-λ are less marked than all of IFNs α and β, regarding bone marrow suppression, probably because fewer cells carry the receptor, thus permitting a more targeted therapy [56].

### Interferon-γ

IFN-γ is a cytokine produced by effector T cells and natural killer cells. It is mainly involved in the development of T helper 1 (Th1) cells [57]. Studies using a HCV replicon system showed that IFN-γ is capable of inhibiting the HCV replication [58]. Similar to other cytokines there is no polymorphism reported in the coding region; but the non-coding region possesses frequent polymorphisms which are implicated in several chronic inflammatory conditions and autoimmune diseases [59,60]. It is reported that the SNP –764G of IFN-γ which is located in the proximal promoter region was strongly associated with SVR in case of HCV infection because the G allele conferred a two to three-fold increase in the promoter activity. Moreover, the G allele also offers stronger binding affinity to the heat shock transcription factor (HSF1) than the C allele at this specific position. Thus, IFN-γ promoter SNP –764G/C is functionally very important which can influence the interferon therapy and may be used as a therapeutic marker for HCV infection [61].

### Mannan-binding lectin

Mannan-binding lectin (MBL) also known as mannose binding protein plays a significant role inthe innate immune system. It is a pathogen recognition receptor [62]. MBL deficiencies are ascribed to a certain extent to three SNPs in the first exon of the gene: MBL2*D (Arg52Cys), B(Gly54Asp) and C (Gly57Glu). These SNPs are collectively marked as O. The major allele at these loci is A. The polymerization of the polypeptide is disturbed by O amino acid which causes low levels of high order oligomeric MBL in plasma [63]. MBL concentration in the serum is also adjusted by two promoter SNPs: MBL2*H/L and X/Y [64,65]. The linkage disequilibrium between the promoter and exon 1 SNPs of MBL produces seven haplotypes which are associated with a decreased level of MBL in the plasma. The frequency of YA/YO genotype was significantly higher in the HCV patients as compared to the controls suggesting that these genotypes are involved in the development of chronic hepatitis C. MBL genotypes XA/XA, XA/YO and YO/YO were associated with a decreased level of MBL in the plasma. The levels of these genotypes were considerably decreased in patients with advanced fibrosis as compared to patients with moderate fibrosis and to the control group. Thus, MBL has not only an important role in the development of chronic hepatitis C, but also an important role in the treatment outcome [63,66].

### Cytotoxic T lymphocyte antigen–4

Cytotoxic T lymphocyte antigen–4 (CTLA4) also referred as cluster of differentiation 152 (CD152) is a protein receptor that is mainly expressed on activated CD4+ and CD8+ T cells [67]. It binds to the ligands B7-1 (CD80) and B7-2 (CD86) and down-regulates the immune system by switching off T cells [68]. There are reported two important transition mutations in CTLA4, C→T at position -318 and G→A at position 49 in exon 1 [69,70]. It is well established by recent findings that these transition mutations of CTLA-4 have a very significant influence on the down regulation of T cells [71]. Sustained responders (SRs) had a higher frequency of 49G alone and were highly associated with -318C in a haplotype. SRs had a higher homozygosity for the -318C-49G haplotype. Moreover, it was also reported that the immune systems of -318C-49G haplotype carriers were declining the viral load more rapidly as compared to other patients. On the other hand, patients which possessed -318 T and 49A showed a reciprocal effect (poor response). There may be several suggested reasons for these effects of CTLA4 polymorphisms. One of these may be the polymorphism of either a single gene or haplotype, which may be responsible for differential expression of a gene. It is also noted that -318 T [72] and 49A [73] are associated with the increased expression of CTLA4. Haplotype -318 T-49A also showed increased expression of CTLA4 [74]. Studies up till now have suggested that polymorphisms at -318C-49G of CTLA4 gene are linked with down regulation of CTLA4 expression that leads to the amplification of T cells response. Alternatively, as CTLA4 acts as a ligand for CD80 and CD86, it may have a role in the developmental pathway of Th1/Th2 cells and may shift their balance [75]–[77]. Another possibility is that these polymorphisms are in linkage disequilibrium with some adjacent markers that have influence on the expression of CTLA4. HCV genotypes and ethnicity have important effects on the treatment outcome. As the reported study was conducted only on white patients infected with HCV genotype 1, these analyses have to be confirmed in other populations effected with different HCV genotypes [78].

### Interleukin 10

Interleukin 10 (IL-10) is a strong immunoregulatory T helper type 2 (Th2) cytokine which is produced by the majority of cells [79]–[81]. Its main function seems to be the regulation of the proliferation and differentiation of different immune cells via influence on the expression of major histocompatibility complex (MHC) class I and class II molecules [82]. It also induces the production of Th1 cytokines [83]. The level of IL-10 varies among individuals and these variations are mostly attributed to polymorphisms in the promoter region of the IL-10 gene [84]. Particularly, 3 SNPs in the promoter region of IL-10 at positions -1082, -819, and -592 are very important [85]. The combination of these 3 SNPs (ATA, ACC, and GCC) is linked to differential expression of IL-10 gene [84]. As IL-10 production affects HCV replication or the host immune system, it is likely to affects the treatment outcome [86]. Many studies have shown that the carriage of the -592A or the -819 T SNP was linked with a sustained virological response. These two sites have a reciprocal effect. The haplotype consisting of the 108-bp IL-10.R microsatellite and -3575 T, -2763C, -1082A, -819 T, -592A were also connected to the treatment response. The IL-10 (108) TCATA haplotype was positively associated with the treatment response. Its frequency was higher in responders than in non-responders while the other haplotype IL-10 (110) TCATA was equally distributed among responders and non-responders suggesting no effect on the treatment response. The most probable reason for this effect of haplotype IL-10 (108) TCATA against HCV infection and treatment response may be the diminished expression of IL-10 [87].

### Interleukin 18

Interleukin 18 (IL-18) also known as interferon (IFN)-γ inducing factor is a pro-inflammatory cytokine which is produced as a pro-IL-18 by the immature dendritic cells, monocytes and macrophages. Pro-IL-18 is activated by caspase1 to generate IL-18 which in turn induces the production of TNF-α and IFN-γ. TNF-α is an anti-inflammatory cytokine which stimulates the production of IL-18 binding protein (IL-18BP) to overcome the increased production of IL-18 in chronic HCV patients for the regulation of inflammation and fibrosis development [88]. Interferon therapy of HCV infection also increases the level of IL-18BP 3–24 folds [89]. The level of IL-18 and its receptors is highly increased in chronic HCV patients and was correlated with a poor treatment outcome [90]. Moreover, an elevated level of IL-18 was also linked with hepatic injury suggesting that it has an important role in liver disease [91]. Earlier studies have shown two important SNPs (-607 C/A and -137 G/C) in the promoter region of IL-18. Lower promoter activity was associated with minor alleles (-607A and -137C) while higher promoter activity was linked with more common alleles (-607C and -137G). These two SNPs are also reported to be associated with Crohns disease [92], cardiovascular diseases [93], HBV [94,95] human immuno deficiency virus (HIV) infection [96] and HCV [97].

### Tumor necrosis factor-related apoptosis inducing ligand receptor 1

Tumor necrosis factor-related apoptosis inducing ligand receptor 1 (TRAIL) also known as CD253 is mostly expressed on effector T cells [98]. It induces apoptosis in virus infected cells [99]. However, there is an increasing evidence to support the dual role of TRAIL in the immune system. It may also play a role in either proviral or antiviral ability. If TRAIL will finally act as a proviral or antiviral is mainly dependent on the overall cytokine situation and also on the type of virus [100]. It has been demonstrated that the SNP, rs4242392 of TRAIL gene is negatively associated with the interferon-based therapy outcome of HIV/HCV co-infected patients [101].

### Transforming growth factor- β

Transforming growth factor (TGF-β) is a multifunctional cytokine. Its main function is to control cellular proliferation and differentiation [102]. Three different isoforms of TGF-β are expressed in mammals; each one is encoded by a unique gene [103]. Among these isoforms, TGF-β1 is the most abundant. There are significant differences between individuals in their ability to produce TGF-β1 because its production is under genetic control. Genetic polymorphism at the codon 10T/C and codon 25G/C of TGF-β1 is linked with differential cytokine secretion. It was reported that treatment response rates to interferon-α therapy in HCV/HIV co-infected patients were enhanced in those patients carrying a 'high-producer’ genotype of TGF-β. The most probable reason for this high response rate may be attributed to partial compensation of HCV NS5A-induced inhibition of TGF-β1 signaling [104]. It was suggested that the polymorphism at codon10T/C of TGF-β1 was linked to 'high-producer’ state but this is still controversial [105].

### Myxovirus resistance protein A

The interferon-induced GTP-binding protein, Myxovirus resistance protein A (MxA), is an abundant ubiquitous cytoplasmic protein which is encoded by the MX1gene [106]. It influences IFN-induced antiviral activities of the host cells against several viruses by acting as mediators for interferons [107]. MxA protein is considered as the most precise surrogate parameter for the action of interferons [108]. The level of mRNA or protein of the MxA gene varied among individuals and it was noted to be significantly associated with the treatment response [109]. The variations of MxA at transcriptional or translational level suggest that its expression is under a strong genetic control. Polymorphism at the nt -88 (G/T) in the promoter region of the MxA gene was linked to IFN response in HCV patients. Patients with the higher SVR rates were mostly MxA-TT [110]. Another study on the Japanese population showed that the rate of G Ghomozygosity was 31% in the SVR patients, significantly lower than in the NR patients. Thus, MxA SNP at the nt -88 affects the expression of MxA protein, and may also affects the treatment response to HCV infection [111].

### Osteopontin

Osteopontin (OPN) is highly phosphorylated sialoprotein. It is an important element of extracellular matrices of bones and teeth [112]. It is also secreted by activated T-lymphocytes, leucocytes and macrophages [113]. This protein plays various physiologic roles in the immune system by interacting with cellular adhesion molecules. Overexpression of this protein was notified in various diseases. OPN has been implicated in the pathogenesis of rheumatoid arthritis (RA). It was found that its level is increased in RA., Its exact role in RA is however still unclear. It was noticed by one group that OPN knocked out mice were protected against RA [114], yet another group of researchers failed to produce the same conclusions [115]. Overexpression of OPN is also noticed in a variety of cancers, including lung, breast, colorectal and stomach cancer. Thus, manipulation of plasma OPN levels provides new opportunities for the treatment of various diseases [116,117]. It was also shown that OPN was essential for the initiation of the Th1 response in mice [118]. SNP at nucleotide the (nt) -443 of osteopontin was associated with hepatitis activity in patients of chronic hepatitis C [119]. Moreover, the response rate was significantly higher in patients with the G/G or G/A alleles at the nt -1748 as compared to those possessing A/A at this position. The response rate was also significantly higher in patients withT/T at the nt -443 than in those with C/C or C/T at this position of OPN [120].

### Low molecular mass polypeptides 7

There is an important role of low molecular mass polypeptides (LMP) in human leukocyte antigen (HLA) class I-restricted antigen presenting systems [121,122]. Genetic variations of LMP gene have a significant influence on the treatment outcome of HCV infection. Genetic variations of LMP gene in 175 HCV patients showed that the frequency of LMP7-K gene in the sustained-responders was significantly higher than in the non-responders. LMP7-K and HCV-RNA quantity were established as an independent factor affecting the outcome of interferon therapy [123].

### Oligoadenylate synthetase 1

IFN therapy against HCV infection is mediated by the stimulation of intracellular antiviral proteins. 2′–5′ oligoadenylate synthetase (OAS) is a critical protein with antiviral activity [124]. It has discriminating activity against several viruses. It is activated by double stranded RNA (dsRNA) to polymerize adenosine triphosphate (ATP) into 2′–5′ linked oligoadenylates which bind and stimulate latent ribonuclease L (RNaseL). RNaseL degrades viral RNA and inhibits protein synthesis [125]. There is a strong genetic control of OAS1 basal activity and the genetic polymorphism at exon 7 splice accepter site (SAS). This makes OAS1 an excellent candidate gene that can importantly affect the host susceptibility to viral infection, disease progression and the treatment response. Recent studies have shown a significant association between the polymorphism at exon 7 SAS of OAS1 gene and the response to interferon therapy in HCV infected patients. It was found that patients possessing the AA genotype at this specific position of the OAS1 gene demonstrated a progressive disease and resistance to the standard treatment [126].

## Patient characteristics

### Age

Patient age is an important factor linked to the treatment response to HCV infection. In general, it is assumed that younger individuals below forty respond better to interferon therapy [127] than older ones. The obvious justification for this association is that aged patients are more likely to have other liver diseases, such as fibrosis and cirrhosis. Moreover, in older age there are more imbalances of cellular, humoral, and innate immunity [128].

### Race

Race is another important host factor that is linked with the treatment outcome [129]. There is increasing evidence that African-American patients respond poorly to interferon therapy compared to non-African-Americans. The range of sustained response for African-Americans was 19%-28% while those for non-African-Americans were 39-52%. Moreover, the viral breakthroughs were also more frequent in African-Americans [130]. The mechanisms behind these observed differences in the treatment response are not properly cleared. It may be due to higher body weight and the HCV genotype 1 prevalence in African-Americans [131]. Another important reason for this low treatment response among the African-American population is the low prevalence of IL28B polymorphism (rs12979860). The prevalence of this SNP among the African-American is only 16% with SVR rate of 47% while among the Caucasians its prevalence is 39% with SVR rate of 81% [132].

### Sex

Initially it was shown that there were significant differences in SVR rate with the female. Female sex is positively associated with SVR [133]. However, large prospective studies showed that there was no effect of sex in achieving SVR [134].

### Obesity

It has been shown that a body mass index (BMI) > 25 kg/m^2^ was linked with fibrosis [135]. Approximately, 30% of HCV patients are obese and they respond poorly to interferon therapy [136,137]. The poor treatment response in these patients is mostly attributed to altered metabolism due to cytokine production by adipocytes. Moreover, there is also a poor absorption of interferon in obese patients [138]. On the contrary, a recent large study showed that there is no significant effect of BMI on the treatment outcome [139]. However, weight loss plays an important role in HCV treatment because it down regulates liver enzymes and the progression of fibrosis [140].

### Alcohol

The use of alcohol induces increased histological activity and fibrosis of the liver. Histological lesions of the liver are accelerated even by moderate use of alcohol in chronic HCV patients [141]. Thus, alcohol intake is related to poor response to interferon therapy [142].

### Insulin resistance

Multiple studies have shown that HCV infection is also linked with insulin resistance [143]. The risk of developing diabetes mellitus is increased up to 11 times in patients with chronic HCV infection [144]. Interferon therapy is also affected by insulin resistance in chronic HCV patients. Insulin resistance causes up-regulation of SOC3 which hinders interferon-mediated signaling pathways [145].

### Hepatic steatosis

Hepatic steatosis accelerates disease progression in HCV patients [146]. There is an involvement of both host and viral factors in steatosis development. In case of HCV genotype 3, steatosis is most commonly induced by the virus while in case of non-genotype 3; it is mostly associated with BMI and central adiposity. Large scale clinical studies have shown that steatosis weakens the treatment response [147].

## Conclusion

Interferon responsiveness is still a main clinical problem in the treatment of HCV. The precise prediction which patient will respond to this therapy is very important, both from the point of the patient care and of the costs. There are both host and viral factors which can significantly predict the probable treatment outcome of HCV patients. HCV genotype other than 1 is the most important predictor of SVR. A number of host factors including SNPs of interferons IL28A, IL28B, IL29, interferon-γ, MBL, IL -10, IL-18, CTLA4, TRAIL, TGF-β, MX1, Osteopontin, LMP7, OAS1 genes, insulin resistance, obesity and ethnicity, have been found to modulate the treatment response. There is still a struggle for discovering new direct-acting inhibitors of HCV that will be used in combination with interferon or without the application of interferon, so further future studies of factors that may predict the treatment outcome of combinational therapies are required.

## Competing interests

The authors declare that they have no competing interests.

## Authors contributions

MI and SM conceived the study and participated in its design. MI searched the literature and drafted the manuscript. SM critically reviewed the manuscript. SM and JA edited the manuscript. JA, MK, MT, HMK, SA helped MI in literature review. SM submitted the review article; she is the PhD supervisor of MI. All the authors read and approved the final manuscript.
